# Risk Factors for No-Reflow in Patients with ST-Elevation Myocardial Infarction Who Underwent Percutaneous Coronary Intervention: A Case-Control Study

**DOI:** 10.1155/2022/3482518

**Published:** 2022-03-10

**Authors:** Ying Yu, Yongquan Wu, Xianyi Wu, Jinwen Wang, Changhua Wang

**Affiliations:** ^1^Department of Cardiology, Beijing Anzhen Hospital, Capital Medical University, Beijing, China; ^2^Daytime Diagnosis and Treatment Department, The Fifth Medical Center of PLA General Hospital, Beijing, China; ^3^Beijing Institute of Heart, Lung, and Blood Vessel Diseases, Beijing, China

## Abstract

**Methods:**

This case-control study retrospectively reviewed the medical data of patients treated with primary percutaneous coronary intervention within 12 h after STEMI onset between January 2010 and January 2013 at the Department of Cardiology of the Beijing Anzhen Hospital.

**Results:**

A total of 902 patients were included in the analysis. The basic characteristics between the reflow and no-reflow groups were similar, except for time-to-hospital admission, heart rate, plasma glucose, high-sensitivity C-reactive protein (hsCRP)/prealbumin (PAB), neutrophil count, intraaortic balloon pump, and aspiration thrombectomy. The multivariable analysis showed that hsCRP/PAB (OR = 1.003, 95% CI: 1.000–1.006, *P*=0.022), neutrophil count (OR = 1.085, 95% CI: 1.028–1.146, *P*=0.003), plasma glucose levels (OR = 1.086, 95% CI: 1.036–1.138, *P*=0.001), diabetes mellitus (OR = 0.596, 95% CI: 0.371–0.958, *P*=0.033), Killip classification >1 (OR = 2.002, 95% CI: 1.273–3.148, *P*=0.003), intraoperative intraaortic balloon pump (IABP) use (OR = 3.257, 95% CI: 1.954–5.428, *P*=0.001), and aspiration thrombectomy (OR = 3.412, 95% CI: 2.259–5.152, *P*=0.001) were independently associated with no-reflow.

**Conclusion:**

hsCRP/PAB, neutrophil count, plasma glucose levels, diabetes mellitus, Killip classification, intraoperative IABP use, and aspiration thrombectomy were independent risk factors for no-reflow in patients with STEMI.

## 1. Introduction

In patients with myocardial ischemia symptoms, ST-elevation myocardial infarction (STEMI) is defined as the combination of persistent ST-segment elevation and the release of biomarkers of myocardial necrosis [1]. Percutaneous coronary intervention (PCI) is the main reperfusion strategy for eligible patients with STEMI [1, 2], but the no-reflow phenomenon is an important cause of adverse PCI outcomes, ventricular remodelling, and poor cardiac function recovery after ischemia–reperfusion [3]. No-reflow significantly increases hospitalization and mortality rates. To date, there is no clear evidence of the reversal of the no-reflow phenomenon, but early monitoring and screening for high-risk patients before PCI could reduce the occurrence of no-reflow events [4, 5]. The specific mechanisms of the no-reflow occurrence are not completely clear but might include distal microvascular embolization and reperfusion-related injury [6]. Inflammatory factors, such as platelets, neutrophils, endothelial cells, tissue factors, and vasoconstrictors, are involved in the process of no-reflow [7, 8].

At present, there is no single effective treatment for no-reflow, so prevention is very important. Identifying patients with the greatest risk is the first step in preventing no-reflow [9, 10]. It is necessary to detect available blood biomarkers and other clinical indicators to predict the risk of no-reflow and reduce the incidence of this phenomenon at an early stage. Therefore, this study obtained the basic data of patients, blood biomarker indices, and specific indices in the process of coronary intervention for the comparative study to provide a clinical basis for the study of no-reflow.

## 2. Methods

### 2.1. Study Design and Population

**Figure 1:**
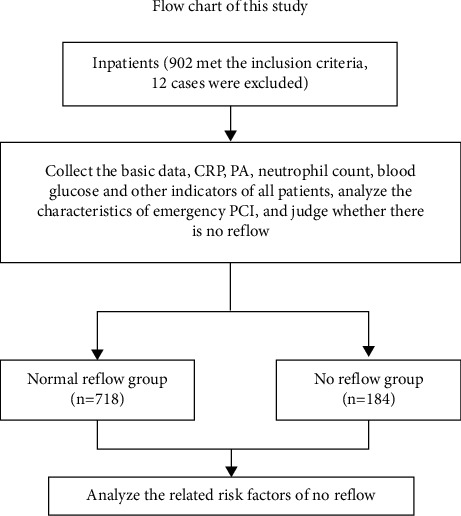
Flowchart.

This case-control study involved patients admitted to the Department of Cardiology of the Beijing Anzhen Hospital within 12 h after the onset of STEMI and treated with primary PCI (pPCI) between January 2010 and January 2013. The inclusion criteria were as follows: 18–85 years of age, STEMI onset within 12 h, and pPCI. The exclusion criteria were as follows: emergency or conventional coronary artery bypass graft (CABG) surgery, thrombolysis, or incomplete medical records (Figure 1). The study was approved by the Ethics Committee of the Beijing Anzhen Hospital (2018066X). The requirement for informed consent was waived because of the retrospective nature of this study.

### 2.2. Data Collection and Definition

The data collected from the medical records included age, sex, smoking, hypertension, diabetes mellitus, prior PCI, preinfarction angina, medication before MI, time-to-hospital admission, physical findings on admission, systolic blood pressure, diastolic blood pressure, heart rate, plasma glucose, hsCRP, PAB, hsCRP/PAB, albumin, hsCRP/albumin, neutrophil count, LDL-C, triglycerides, Killip classes, treatment before/during procedure, glycoprotein IIb/IIIa inhibitor, intraaortic balloon pump, angiography, multivessel disease, infarct-related artery, left main artery, left anterior descending artery, left circumflex artery, right circumflex artery, aspiration thrombectomy, after dilation, stent diameter, and total stent length.

STEMI was defined as the presence of new ST-elevation at the J-point in two contiguous leads with the following cutoff points: ≥0.25 mV in men <40 years old, ≥0.2 mV in men ≥40 years old, ≥0.15 mV in women in leads V2-V3, and/or ≥0.1 mV in other leads, or presumed new left bundle-branch block; and creatine kinase-MB (CK-MB) levels above the normal levels in patients who had prolonged chest pain lasting for ≥30 min [11]. Cardiac symptoms that persisted for ≥30 min within 48 h before the onset of infarction were defined as preinfarction angina.

The time of the first demonstration of the presence and severity of heart failure was categorized according to the Killip classification [12, 13]. The perfusion status of the infarct-related artery was evaluated based on the myocardial blush grade (MBG) [14, 15]. No-reflow angiography was defined as thrombolysis in myocardial infarction (TIMI) flow grade <3 or 3 with an MBG of 0-1 [16]. The two cardiologists who evaluated the presence of reflow made their evaluations independently. A consensus had to be reached in cases of disagreement.

### 2.3. Statistical Analysis

Statistical analysis was performed using SPSS 19.0 (IBM, Armonk, NY, USA). Continuous data were tested for normal distribution using the Kolmogorov–Smirnov test and are shown as the means ± standard deviations (SD) or medians (25^th^–75^th^ percentiles). Continuous variables were analysed using the Mann–Whitney *U* test or Student's *t*-test. Categorical variables are presented as *n* (%) and were analysed using the chi-square test. Univariable and multivariable stepwise logistic regression analyses were performed with adjustment for diabetes mellitus, age, hypertension, smoking, preinfarction angina, prior PCI, time from pain to pPCI, Killip class, use of cardiovascular medication before STEMI, pPCI as reperfusion therapy, physical findings, electrocardiographic findings, stenting methods, postdilation, stents, stent diameter, thrombolysis before PCI, intraaortic balloon pump (IABP) use, tirofiban use, and aspiration thrombectomy. Candidate variables with *P* < 0.20 were eligible for conditional stepwise multivariable logistic regression. A threshold with a significant odds ratio (OR) for predicting no-reflow was identified using a threshold of *P* < 0.05.

## 3. Results

A total of 914 patients were screened. One patient was excluded due to thrombolysis, six due to CABG, and five due to incomplete data. Finally, 902 patients were included for analysis. Among them, 184 (20.4%) had no-reflow. The basic characteristics between patients with no-reflow and reflow tended to be similar, except for time-to-hospital admission (*P* < 0.001), heart rate (*P* < 0.001), plasma glucose (*P* < 0.001), hsCRP (*P*=0.004), PAB levels (*P*=0.008), hsCRP/PAB (*P* < 0.001), hsCRP/albumin (*P*=0.028), neutrophil count (*P* < 0.001), intraaortic balloon pump (*P* < 0.001), and aspiration thrombectomy (*P* < 0.001) (Table 1).

Univariable analyses showed that heart rate, plasma glucose levels, hsCRP levels, PAB levels, hsCRP/PAB, neutrophil count, Killip classification >1, intraoperative IABP use, infarct-related artery, time-to-hospital admission, and aspiration thrombectomy were potential risk factors for no-reflow (Table 2). Furthermore, the multivariable analysis showed that hsCRP/PAB (OR = 1.003, 95% CI: 1.000–1.006, *P*=0.022), neutrophil count (OR = 1.085, 95% CI: 1.028–1.146, *P*=0.003), plasma glucose levels (OR = 1.086, 95% CI: 1.036–1.138, *P*=0.001), diabetes mellitus (OR = 0.596, 95% CI: 0.371–0.958, *P*=0.033), Killip classification >1 (OR = 2.002, 95% CI: 1.273–3.148, *P*=0.003), intraoperative IABP use (OR = 3.257, 95% CI: 1.954–5.428, *P*=0.001), and aspiration thrombectomy (OR = 3.412, 95% CI: 2.259–5.152, *P*=0.001) were independent risk factors for no-reflow (Table 3).

## 4. Discussion

Acute myocardial infarction is one of the most serious clinical manifestations of coronary heart disease. The main international heart guidelines still recommend PCI as the first choice for reperfusion therapy in patients with acute myocardial infarction [2]. However, no-reflow can occur in patients with acute ST-segment elevation myocardial infarction after PCI, which seriously affects the short-term and long-term prognoses [17]. No-reflow is a complication that increases the incidence of adverse cardiac outcomes and hospital deaths [18]. At present, ischemia–reperfusion injury is the main mechanism of no-reflow and is the result of the joint actions of platelets, neutrophils, endothelial cells, and tissue factors [19]. Reperfusion injury causes inflammation and immune activation, leading to complex interactions between inflammatory mediators, platelets, neutrophils, and oxygen free radicals [20–22]. The results of this study showed that hsCRP/PAB, neutrophil count, blood glucose level, diabetes, Killip classification, IABP, and thrombus removal were independent risk factors for reflow in STEMI patients.

C-reactive protein (CRP) is a classic marker of inflammation and the most reliable inflammatory marker of atherosclerosis [23]. Karabag et al. [24] showed that high-sensitivity CRP (hsCRP) can predict no-reflow. Clinical and experimental studies have shown that blood hsCRP levels are an independent risk factor without reflow [25, 26]. In this study, the CRP levels were significantly associated with no-reflow after PCI. Prealbumin (PAB) is a negative acute phase reactive protein synthesized by the liver that is closely related to the occurrence and development of atherosclerosis [27]. Recent studies have shown that the hsCRP/PAB ratio can predict the prognosis of patients in various situations, such as acute renal injury [28], parenteral nutrition [29], fistula [30], and organ dysfunction [31]. Zhang et al. [32] examined the severity of acute coronary syndrome using PAB and hsCRP/PAB and found that PAB and hsCRP/PAB were significantly correlated with the Gensini score. Wang et al. [33] reported that the hsCRP/PAB ratio was associated with major adverse coronary events after STEMI. In this study, the hsCRP/PAB ratio was independently correlated with no-reflow after PCI, indicating that it was correlated with a poor prognosis for coronary artery disease. The possible mechanism was related to the expansion of the local infarct area, aggravation of the inflammatory response and reperfusion injury.

Hyperglycemia significantly affects the prognosis of STEMI patients. Yildiz et al. [34] evaluated TFC (thrombolytic frame count of myocardial infarction) of 121 STEMI patients after pPCI. It was found that TFC of hyperglycemia patients increased significantly, and the incidence of no-reflow in the hyperglycemia group was higher than that in the normal blood glucose group. Multiple linear regression analysis showed that admission hyperglycemia was an independent predictor of high TFC. Mone et al. [35] found that the risk of stent restenosis after pPCI was significantly increased in STEMI patients with hospitalized hyperglycemia.

This study found that there was a significant correlation between hyperglycemia and no-reflow, which was consistent with previous studies [36]. The underlying pathophysiological mechanisms that may lead to the adverse prognostic effects of hyperglycemia are unclear, but the following common understanding exists. First, hyperglycemia will aggravate leukocyte blockage in microcirculation, and acute hyperglycemia will increase the level of intercellular adhesion molecule-1 or P-selectin [37]. This will increase leukocyte blockage in capillaries and may further lead to the no-reflow phenomenon. Hyperglycemia may also increase thrombosis. A clinical study showed that microthrombosis in capillaries plays a key role in no-reflow after AMI. Hyperglycemia is an independent predictor of platelet-dependent thrombosis, and ischemic preconditioning is an independent predictor of the no-reflow phenomenon [38]. Hyperglycemia may weaken the effect of ischemic preconditioning by reducing the activation of potassium channels regulated by mitochondrial adenosine triphosphate [39]. This would thereby reduce collateral circulation to risk areas, resulting in greater myocardial injury before reperfusion that is followed by no-reflow. Finally, hyperglycemia may be related to reperfusion injury. In the rat heart, diabetic blood enhances myocardial reperfusion injury by enhancing cell adhesion to capillaries and the generation of free radicals [40]. Previous studies have shown that the incidence of ST-segment reelevation after coronary reperfusion in the hyperglycemia group is higher, suggesting the occurrence of myocardial reperfusion injury.

At present, some studies have discussed how to reduce thrombus load, improve endothelial cell function, and expand coronary artery by local administration of IIb/IIIa inhibitor through intracoronary route [41] and intravenous infusion of adenosine [42], so as to improve myocardial perfusion, increase coronary blood flow, reduce the incidence of no-reflow, and improve the long-term prognosis of STEMI patients with hyperglycemia, However, large sample research and in-depth discussion are still needed.

This study found that 184 cases (20.4%) had no-reflow. The neutrophil count in the no-reflow group was significantly higher than that in the normal reflow group. Neutrophil count was an independent predictor of no-reflow. A previous meta-analysis [43] showed that the high and middle neutrophil count groups had a higher risk of no-reflow than the low neutrophil count group. The underlying mechanism of neutrophil participation in no-reflow is complex. Ischemic injury of cardiomyocytes manifests as cardiomyocyte swelling and interstitial oedema. Pathological changes in cardiomyocytes increase the compression of intramural vessels and induce neutrophil blockage and activation in coronary microcirculation. Oxygen free radicals released by activated neutrophils contribute to endothelial injury and reperfusion injury. During reperfusion, due to the excessive production of reactive oxygen species, neutrophils adhere to endothelial cells and then activate NF-*κ*B cascade. The structural lumen obstruction of microvessels is caused by microaggregates formed by neutrophils and platelets, which aggravate reperfusion injury [44]. In addition, due to the increase in vascular permeability, neutrophil infiltration in vulnerable myocardium enhances interstitial oedema and extravascular mechanical compression, resulting in the no-reflow pathological process [45].

In this study, we found that a Killip classification >1 was associated with no-reflow. Patients without reflow had a higher Killip grade, which is consistent with the results of Zhou et al. [46]. The higher Killip grade in patients without reflow may be related to larger infarct size and reduced coronary perfusion pressure. The decrease in coronary artery pressure accelerates the blockage of microvessels by neutrophils, resulting in no-reflow.

This study has some limitations. The sample size was relatively small and limited to a single hospital. Furthermore, the evaluation of no-reflow after pPCI was visually assessed based on angiograms without echocardiography and cardiac magnetic resonance examinations.

## 5. Conclusion

HsCRP/PAB, neutrophil count, plasma glucose levels, diabetes mellitus, Killip classification, intraoperative IABP use, and aspiration thrombectomy are the independent risk factors for no-reflow after pPCI.

## Figures and Tables

**Table 1:** Clinical, angiographic, and procedural data for no-reflow.

Characteristics	Reflow (*n* = 718)	No-reflow (*n* = 184)	*P*
Clinical data			
Age (years)	57 ± 11	59 ± 11	0.051
Male sex, *n* (%)	591 (82.3)	147 (79.9)	0.448
Hypertension, *n* (%)	367 (51.1)	88 (47.8)	0.426
Diabetes mellitus, *n* (%)	192 (26.7)	43 (23.4)	0.353
Smoking, *n* (%)	488 (68.0)	126 (68.5)	0.923
Prior PCI, *n* (%)	28 (3.9)	11 (6.0)	0.216
Preinfarction angina, *n* (%)	420 (58.5)	101 (54.9)	0.377

Medication before MI, *n* (%)			
Aspirin	62 (8.6)	18 (9.8)	0.625
ACEI	99 (13.8)	21 (11.4)	0.397
*β*-Blocker	59 (8.2)	10 (5.4)	0.205
Calcium channel blockers	177 (24.7)	42 (22.8)	0.606
Statin	24 (3.3)	5 (2.7)	0.668

Physical findings on admission			
Systolic blood pressure (mmHg)	120 ± 21	118 ± 21	0.209
Diastolic blood pressure (mmHg)	75 ± 13	75 ± 13	0.992
Heart rate (bpm)	75 (66, 82)	78 (70, 92)	<0.001
Time-to-hospital admission (hours)	8.0 (5.0, 24.0)	7.0 (4.0, 12.0)	<0.001

Laboratory indicators			
Plasma glucose (mmol/L)	8.08 ± 4.83	10.11 ± 5.48	<0.001
hsCRP (mg/L)	7.21 (2.89, 13.79)	9.36 (3.74, 18.57)	0.004
PAB (g/L)	0.25 ± 0.06	0.23 ± 0.06	0.008
hsCRP/PAB	29.72 (11.30, 65.96)	38.18 (14.97, 99.08)	<0.001
Albumin (g/L)	41.43 ± 7.53	41.19 ± 7.58	0.701
hsCRP/albumin	0.27 ± 0.37	0.33 ± 0.31	0.028
Neutrophil count (×10^9^/L)	8.14 ± 3.25	10.01 ± 3.33	<0.001
LDL-C (mmol/L)	3.40 ± 12.62	2.93 ± 0.92	0.614
Triglycerides (mmol/L)	2.04 ± 1.74	2.13 ± 4.60	0.655
Killip classes, *n* (%)			0.064
1	191 (26.6)	35 (19.0)	
2	497 (69.2)	140 (76.1)	
3	19 (2.6)	3 (1.6)	
4	11 (1.5)	6 (3.3)	

Treatment before/during procedure, *n* (%)			
Glycoprotein IIb/IIIa inhibitor	101 (14.1)	17 (9.2)	0.083
Intraaortic balloon pump	43 (6.0)	44 (23.9)	<0.001

Angiography			
Multivessel disease	202 (28.1)	46 (25.0)	0.396
Infarct-related artery, *n* (%)			0.148
Left main artery	2 (0.3)	1 (0.5)	
Left anterior descending artery	363 (50.6)	103 (56.0)	
Left circumflex artery	110 (15.3)	17 (9.2)	
Right circumflex artery	243 (33.8)	63 (34.2)	

Procedure			
Aspiration thrombectomy, *n* (%)	338 (47.1)	144 (78.3)	<0.001
After dilation, *n* (%)	375 (52.2)	84 (45.7)	0.111
Stent diameter (mm)	2.9 ± 0.7	3.0 ± 0.9	0.334
Total stent length (mm)	32.7 ± 15.5	33.1 ± 15.9	0.753

Continuous data are shown as means ± standard deviations (SD) or median (25^th^–75^th^ percentiles). PCI, percutaneous coronary intervention; MI, myocardial infarction; ACEI, angiotensin-converting enzyme inhibitor; hsCRP, high-sensitivity C-reactive protein; PAB, prealbumin; LDL-C, low-density lipoprotein cholesterol.

**Table 2:** Univariable analysis for no-reflow.

Variables	Univariable analysis	
	OR (95% CI)	*P*
Clinical data		
Age	1.015 (1.000–1.030)	0.052
Male	1.171 (0.779–1.762)	0.448
Hypertension	1.141 (0.825–1.577)	0.426
Diabetes mellitus	1.197 (0.819–1.749)	0.353
Smoking	0.983 (0.694–1.393)	0.923
Prior PCI	0.638 (0.312–1.307)	0.220
Preinfarction angina	1.158 (0.836–1.605)	0.377

Medication before MI		
Aspirin	0.872 (0.502–1.513)	0.625
ACEI	1.241 (0.752–2.050)	0.398
*β*-Blocker	1.558 (0.781–3.108)	0.208
Calcium channel blockers	1.106 (0.754–1.624)	0.606
Statin	1.238 (0.466–3.290)	0.669
Time-to-hospital admission	0.985 (0.976–0.995)	0.002

Physical findings on admission		
Systolic blood pressure	0.995 (0.987–1.003)	0.209
Diastolic blood pressure	1.000 (0.987–1.013)	0.992
Heart rate	1.026 (1.016–1.037)	<0.001

Laboratory indicators		
Plasma glucose	1.088 (1.042–1.135)	<0.001
hsCRP	1.024 (1.009–1.039)	0.001
PAB	0.026 (0.002–0.385)	0.008
hsCRP/PAB	1.005 (1.003–1.008)	<0.001
Albumin	0.995 (0.973–1.019)	0.701
hsCRP/albumin	1.551 (0.963–2.499)	0.071
Neutrophil count	1.172 (1.117–1.229)	<0.001
LDL-C	0.989 (0.930–1.052)	0.733
Triglycerides	1.013 (0.958–1.070)	0.659
Killip classes >1	0.648 (0.433–0.970)	0.035

Treatment before or during the procedure		
Glycoprotein IIb/IIIa inhibitor	1.608 (0.936–2.764)	0.086
Intraaortic balloon pump	0.203 (0.128–0.320)	<0.001

Angiography		
Multivessel disease	1.174 (0.710–1.702)	0.396
Infarct-related artery	0.557 (0.325–0.954)	0.033

Procedure		
Aspiration thrombectomy	0.247 (0.169–0.361)	<0.001
After dilation	1.302 (0.940–1.801)	0.112
Stent diameter	1.114 (0.894–1.388)	0.334
Total stent length	1.002 (0.991–1.012)	0.752

PCI, percutaneous coronary intervention; MI, myocardial infarction; ACEI, angiotensin-converting enzyme inhibitor; hsCRP, high-sensitivity C-reactive protein; PAB, prealbumin; LDL-C, low-density lipoprotein cholesterol.

**Table 3:** Multivariable analysis for no-reflow.

Variables	Multivariable analysis	
	OR (95% CI)	*P*
Diabetes mellitus	0.596 (0.371–0.958)	0.033
Heart rate	1.019 (1.007–1.031)	0.002
Plasma glucose	1.086 (1.036–1.138)	0.001
hsCRP/PAB	1.003 (1.000–1.006)	0.022
Neutrophil count	1.085 (1.028–1.146)	0.003
Killip classes >1	2.002 (1.273–3.148)	0.003
Intraaortic balloon pump	3.257 (1.954–5.428)	<0.001
Procedure		
Aspiration thrombectomy	3.412 (2.259–5.152)	<0.001

hsCRP, high-sensitivity C-reactive protein; PAB, prealbumin.

## Data Availability

The data used to support the findings of this study are available from the corresponding author upon request.
